# ﻿Diversity and conservation of terrestrial vertebrates (birds, mammals, and reptiles) of Sierra Cucapá, Mexicali, Baja California, Mexico

**DOI:** 10.3897/zookeys.1088.76134

**Published:** 2022-02-28

**Authors:** Rafael Villegas-Patraca, José Luis Aguilar-López, Julio César Hernández-Hernández, Oscar Muñoz-Jiménez

**Affiliations:** 1 Unidad de Servicios Profesionales Altamente Especializados (USPAE), Instituto de Ecología, A.C., Carretera Antigua Xalapa-Coatepec esquina camino a Rancho Viejo No. 1, Fraccionamiento Briones, C. P. 91520 Coatepec, Veracruz, México Unidad de Servicios Profesionales Altamente Especializados (USPAE) Veracruz Mexico

**Keywords:** citizen science, databases, fieldwork, natural protected areas system, species composition

## Abstract

Knowledge about the biodiversity of Baja California has been obtained mainly from natural protected areas (NPAs), while some unprotected natural areas have been poorly studied. The Sierra Cucapá in the northeast of the peninsula is one example. The objectives of this study are 1) to integrate existing knowledge of bird, mammal, and reptile diversity in Cucapá from public databases, citizen science platforms, and information generated from fieldwork, 2) to identify the spatial distribution of records in the study area, 3) to compare the composition of vertebrate species of Cucapá with that of NPAs of northern part of the peninsula, and 4) to assess the biological conservation value of Cucapá. We obtained records of 150 species of native vertebrates (102 birds, 34 mammals, and 14 reptiles) of which 10 species of birds, four mammals, and seven reptiles are included in a risk extinction category. The different sources of information contributed in a complementary way to the species inventories. Large areas in western and northern Cucapá lack records. The total difference in species composition between Cucapá and nearby NPAs ranged between 58 and 69% for birds, 61 and 79% for mammals, and 69 and 87% for reptiles. The species richness of Cucapá, its particular species composition, the presence of species in risk extinction categories, and the number and size of unexplored areas indicate that this area represents an opportunity for biological conservation in the northern part of the Peninsula. This work provides compelling data for the protection of Cucapá.

## ﻿Introduction

The Baja California Peninsula (hereafter the Peninsula) in northwestern Mexico is a biologically important region with a remarkable species richness and high amount of endemism in various biological groups (Rojas-Soto et al. 2003; Riemann and Ezcurra 2005; Ramírez-Acosta et al. 2012). For example, 514 species of birds (Erickson et al. 2013), 70 mammals (Guevara-Carrizales et al. 2016), and 99 reptiles (Hollingsworth et al. 2015) have been recorded. However, biological diversity on the Peninsula has been evaluated mostly in natural protected areas (NPAs), with gaps in or knowledge of species richness especially in non-protected areas.

The NPAs system in the northern part of the Peninsula includes eight areas covering two main ecosystems types: arid environments under 100 m above sea level (a.s.l.) in the extreme northeast, and coniferous forests between 1500 and 1900 m a.s.l. in the Sierra de Juárez. Biological information on certain groups of terrestrial vertebrates is available for some of these NPAs under government administration (e.g. CONANP 2007, 2011; Guevara-Carrizales et al. 2013; Hinojosa-Huerta et al. 2013). However, some NPAs under community or private management, as well as unprotected natural areas, lack species inventories, making difficult to evaluate their potential for biological conservation and consequently limiting the possibility of including unprotected areas in formal protection schemes.

The Sierra Cucapá (hereafter Cucapá) is an unprotected mountainous massif in the northeast of the Peninsula covering 36400 ha and extending approximately 60 km with a northwest–southeast orientation. Cucapá is known to harbor endemic species of flora and fauna (Grismer 2002; Webb and Salazar-Ceseña 2011) and is located in the proximity of several NPAs under different types of administration (government, private, and community). However, its biological diversity has not been characterized, and its potential contribution to the biological conservation of the region, or its complementarity to the existing NPAs system, has not been evaluated (Riemann and Ezcurra 2005; Lovich et al. 2009; Martínez et al. 2016). Northeast of Cucapá lies the city of Mexicali with a population > 1 million and considerable agricultural production (http://www.inegi.gob.mx). Furthermore, the region is experiencing a growing industrial activity that includes renewable energy generation (Alemán-Nava et al. 2014). Thus, human activities around Cucapá represent a threat to its biodiversity.

Here we present the first comprehensive summary of the biological diversity of three groups of terrestrial vertebrates in Cucapá and the first analysis of its complementarity to the NPAs system of the northern Peninsula. Our objectives are 1) to integrate existing knowledge of bird, mammal, and reptile diversity in Cucapá combining information from public databases, citizen science platforms, and information generated from fieldwork, 2) to identify the spatial distribution of records into the study area, 3) to compare the species composition of birds, mammals, and reptiles of Cucapá with that of NPAs of northern part of the Peninsula, and 4) to assess the biological conservation value of Cucapá.

## ﻿Methods

### ﻿Study area

**Figure 1.**
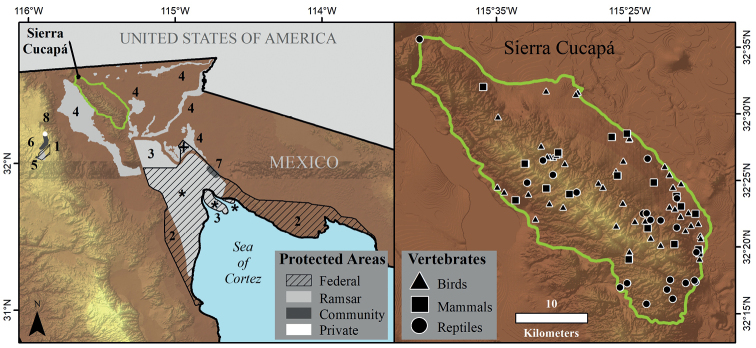
Location of protected natural areas in northern Baja California: government NPAs 1) Parque Nacional Constitucion de 1857, 2) Reserva de la Biosfera Alto Golfo de California y Delta del Río Colorado; Ramsar Sites 3) Humedales del Delta del Río Colorado, 4) Sistema de Humedales Remanentes del Río Colorado, 5) Laguna Hanson; Community NPAs 6) Rancho Rodeo del Rey, 7) El Doctor, and Private NPAs 8) Rancho Rodeo del Rey. * indicates the overlap zone between NPAs 2 and 3, + indicates the overlap zone between NPAs 2 and 4.

Cucapá is located within the Gulf of Baja California Extensional Province, between the latitudinal and longitudinal ranges of 32°15'–32°39'N, 115°19'–115°47'W, respectively (Fig. 1), and at an elevation range of ~0–1,030 m a.s.l. The climate is dry semi-warm, with low rainfall and surface water runoff less than 10 mm. The vegetation type is microphilous desert scrub, composed of shrubs such as the creosote bush (*Larreatridentata*) and burrobush (*Ambrosiadumosa*), as well as large areas of desert ironwood (*Olneyatesota*) and California barrel cactus (*Ferocactuscylindraceus*). Cucapá is bordered to the north by the Mexicali Valley, to the south by the Sierra El Mayor, to the west by the Laguna Salada basin (a dry ephemeral lake), and to the east by the geothermal field of the Cerro Prieto Volcano (Chora-Salvador 2003).

### ﻿Data collection

We compiled records of bird, mammal, and reptile species in Cucapá from three sources 1) the Global Biodiversity Information Facility database (GBIF; https://www.gbif.org), 2) citizen science platforms iNaturalist (https://www.inaturalist.org), and eBird (https://www.ebird.org/), and 3) fieldwork.

Both citizen science platforms were accessed between May and June 2020 and, in the case of iNaturalist, we only considered records tagged as having research quality that were accompanied with photographs in which species identity could be verified. We conducted fieldwork in four different periods (9–24 October 2017, 26 February–12 March 2018, 21 February–4 March 2019, 4 March– 8 June 2019), and, when a method involved captures (see below), all individuals were released after being measured, weighed, photographed, and identified to species.

### ﻿Fieldwork

#### Bird sampling

During three seasons (autumn, winter, and spring), we obtained visual and acoustic records of birds along 43 linear transects of variable distance (Bibby et al. 1992; Ralph et al. 1996) conducted within the first four hours after sunrise. Our total sampling effort was of 172 hours and 358.1 km traveled; however, we also considered for analysis ad libitum records outside the transects. Additionally, six and eight mist nets (12 × 2.5 m) were placed at each of two stations monitored during the spring, which remained active from 06:00 to 10:00 h, and these nets were checked on average every 30 min (but the time interval between net checks was reduced when the number of captured individuals increased). Our total mist-netting effort was of 56 net-h.

#### Mammal sampling

We sampled small mammal diversity at 15 sites. At each site, we placed four parallel lines 10 m apart with five Sherman traps each. We baited the traps with a mixture of oats and vanilla, placed them at sunset (18:00 h) and checked them at dawn (06:00 h), for a total sampling effort of 1200 trap-nights. We recorded medium and large mammal species (i.e. > 500 g, Carrillo et al. 2000; Pozo-Montuy et al. 2019) using six camera traps (CuddeLink Long Range IR) per sampling period (Reid 2009). We placed the cameras at sites with indication of mammal presence (e.g. footprints, feces). The cameras were active 24 h/d for 13 d every sampling period, and we programmed them to take five photographs and a 10-s video at 1-min intervals. The total sampling effort with this method was of 320 trap-d. We also obtained direct observations of mammals or of their presence (e.g. tracks, excreta, remains), through 18 diurnal transects (1–3 km long) that we conducted every sampling period between 08:00–12:00 h, for total sampling effort of 72 h. We placed transects at suitable sites with different vegetation types (Rudran et al. 1996; Gallina-Tessaro and López-González 2011).

We recorded the vocal signature of bats along six 2.5-km transects using a Song Meter SM2BAT (Wildlife Acoustics, Inc.; sampling frequency 384 kHz) coupled with an ultrasonic microphone SMX-U1. At each transect we recorded for 90 min starting at sunset (18:00 h), for a total sampling effort of 540 min of recordings. We analyzed the recordings with Batsound 3.1.0 (Pettersson Elektronik AB, Uppsala, Sweden), and identified species comparing the obtained sonograms with those in the literature (Rizo-Aguilar 2008; Orozco-Lugo et al. 2013).

#### Reptile sampling

We established 55 plots (500 × 10 m) to sample reptile diversity. At each plot, one person searched for reptiles using the time-constrained technique (Crump and Scott 1994), surveying typical microhabitats used by species (e.g. under rocks, fallen logs, and cavities). We carried out three searches per day, in the morning (08:00 – 11:00 h), evening (16:00 – 19:00 h), and night (20:00 – 23:00 h), with a total sampling effort of 495 person-hours.

### ﻿Data analysis

We compiled records from all sources in a database that we updated according to recent taxonomic changes. We only included records identified to the species level that also included its precise location (i.e. geographic coordinates). We checked the currently known geographic distribution of each species in the database and filtered out those records that did not overlap with it. We consulted taxonomic information and geographic distribution ranges on specialized platforms (http://www.reptile-database.org/, Uetz et al. 2020; https://www.iucnredlist.org/, IUCN 2020; http://www.ebird.org) and bibliographic sources (Howell and Webb 1995; Rodríguez-Robles and Jesús-Escobar 2000; Grismer and Hollingsworth 2001; Grismer 2002; Reid 2006; Schulte II et al. 2006; Leaché and Mulcahy 2007; McGuire et al. 2007; González-Bernal 2008; Medellín et al. 2008; Mulcahy 2008; Pyron and Burbrink 2009; Ceballo and Arroyo-Cabrales 2012; Sibley 2014; Cox et al. 2018; O’Connell and Smith 2018; Bradley et al. 2019). We mapped the selected records with ArcGis 10.2.2 (ESRI 2014).

We obtained the conservation status of species from the list of species at risk of extinction (NOM-059-SEMARNAT-2010) published by the Ministry of the Environment and Natural Resources of Mexico (SEMARNAT 2010), which includes the following categories: Threatened (A; Amenazada), Subject to Special Protection (Pr; Sujeta a Protección Especial), Endangered (P; En Peligro de Extinción) and Probably Extinct in the Wild (E; Probablemente Extinta en el Medio Silvestre); and from the Red List of the International Union for Conservation of Nature (IUCN 2020), which includes the categories Vulnerable (VU) Endangered (EN), and Critically Endangered (CR).

We compiled records of bird, mammal, and reptile species in eight NPAs located in the north part of Baja California state, within a 63 km radius from Cucapá (Fig. 1) using GBIF, citizen science platforms, and specialized literature, giving the data the same treatment as described above. The NPAs included in the study are 1) Parque Nacional Constitución de 1857 (CONANP 2011), 2) Reserva de la Biosfera Alto Golfo de California y Delta del Río Colorado “RBAGC” (Guevara-Carrizales et al. 2013; Hinojosa-Huerta et al. 2013); Ramsar sites 3) Humedales del Delta del Río Colorado “HDRC” (Guevara-Carrizales et al. 2013), 4) Sistema de Humedales Remanentes del Río Colorado “SHRRC”, 5) Laguna Hanson; community NPAs 6) Rancho Rodeo del Rey, 7) El Doctor (Guevara-Carrizales et al. 2013), and private NPAs 8) Rancho Rodeo del Rey. For each group of vertebrates, we conducted pairwise comparisons of species composition between all areas, Cucapá included, except for El Doctor and the private Rodeo del Rey, for which we did not obtain species records for any group. Similarly, for the community NPA Rodeo del Rey, we only obtained bird species records, and hence we could not compare its mammal and reptile diversity with the other areas. We followed Baselga and Orme (2012) to estimate total species dissimilarity between pairs of sites with the Jaccard index (β_jac_) and its two components, species turnover (β_jtu_) and species nestedness (β_jne_), expressed with the formula:

$\beta_{jac}=\beta_{jtu}+\beta_{jne}=\frac{b+c}{a+b+c}=\frac{2b}{2b+a}+\left(\frac{c-b}{a+b+c}\right)\left(\frac{a}{2b+a}\right)$

where *a* is the number of species shared between two sites, *b* the number of unique species from the poorest site, and *c* the number of unique species at the richest site. The total dissimilarity value ranges from 0 (when all species are shared) to 1 (when there are no shared species). The analysis was carried out using the language and environment for statistical computing R version 3.1.3 (Core Team 2015) and the betapart package (Baselga and Orme 2012).We only considered native species for analysis.

## ﻿Results

We recorded a total of 150 species of vertebrates for Cucapá: 102 species of birds, taxonomically grouped in 15 orders, 38 families and 83 genera; 34 species of mammals, grouped into six orders, 14 families and 26 genera, and 14 species of reptiles, belonging to one order, five families and 12 genera (Tables 1, 2). Additionally, we recorded six species of exotic birds, but we did not include those in the analysis (Table 1). The best-represented families (by number of species) are Passerellidae in birds (11 spp.), Heteromyidae in mammals (eight spp.), and Phrynosomatidae in reptiles (nine spp.). Sixteen, seven, and three bird, mammal, and reptile families, respectively, were represented by a single species (Table 1).

**Table 1.** List of species and conservation status of birds, mammals and reptiles of Cucapá. The source of record: 1 = GBIF with collected specimens, 2 = iNaturalist and eBird observations (the second, only for birds), 3 = fieldwork. The risk extinction categories from NOM-059-SEMARNAT-2010 are A = Threatened, Pr = Subject to Special Protection, P = Endangered, E = Probably Extinct in the Wild. The risk extinction categories from the IUCN Red List are: LC = Least Concern, NT = Near Threatened, VU = Vulnerable. * = exotic species.

Class/order/suborder/family/species	Source	NOM-059	IUCN
**Class AVES**			
**Order Anseriformes**			
**Anatidae**			
*Anasplatyrhynchos*	3	A	LC
*Spatuladiscors*	3		LC
**Order Galliformes**			
**Odontophoridae**			
*Callipeplagambelii*	1,2,3		LC
**Phasianidae**			
Phasianuscolchicus*	2,3		LC
**Order Columbiformes**			
**Columbidae**			
*Columbinainca*	2		LC
Columbalivia*	2,3		LC
Streptopeliadecaocto*	2,3		LC
*Zenaidaasiatica*	2,3		LC
*Zenaidamacroura*	2,3		LC
**Order Cuculiformes**			
**Cuculidae**			
*Geococcyxcalifornianus*	2,3		LC
**Order Caprimulgiformes**			
**Caprimulgidae**			
*Chordeilesacutipennis*	1,2,3		LC
*Phalaenoptilusnuttallii*	2		LC
**Order Apodiformes**			
**Apodidae**			
*Aeronautessaxatalis*	3		LC
**Trochilidae**			
*Calypteanna*	2,3		LC
*Calyptecostae*	2,3		LC
*Selasphorusrufus*	2,3		NT
**Order Gruiformes**			
**Rallidae**			
*Fulicaamericana*	3		LC
*Gallinulagaleata*	3		LC
*Porzanacarolina*	2,3		LC
**Order Charadriiformes**			
**Recurvirostridae**			
*Himantopusmexicanus*	2		LC
**Charadriidae**			
*Charadriusvociferus*	2,3		LC
**Scolopacidae**			
*Calidrismauri*	2		LC
*Calidrisminutilla*	2,3		LC
*Limnodromusscolopaceus*	2		LC
*Numeniusamericanus*	3		LC
*Tringamelanoleuca*	2		LC
**Laridae**			
*Larusargentatus*	2		LC
*Laruscalifornicus*	2		LC
*Larusdelawarensis*	2,3		LC
*Larusfuscus*	2		LC
*Larusglaucescens*	2		LC
**Order Pelecaniformes**			
**Ardeidae**			
*Ardeaalba*	2,3		
*Ardeaherodias*	2,3		LC
Bubulcusibis*	2,3		LC
*Egrettathula*	3		LC
**Threskiornithidae**			
*Plegadischihi*	2,3		LC
**Order Cathartiformes**			
**Cathartidae**			
*Cathartesaura*	2,3		LC
**Order Accipitriformes**			
**Accipitridae**			
*Accipitercooperii*	2,3	Pr	LC
*Accipiterstriatus*	2,3	Pr	LC
*Buteojamaicensis*	2,3	Pr	LC
*Buteolineatus*	3	Pr	LC
*Buteoregalis*	2	Pr	LC
*Circushudsonius*	2,3		LC
*Elanusleucurus*	2		LC
*Parabuteounicinctus*	1	Pr	LC
**Order Strigiformes**			
**Tytonidae**			
*Tytoalba*	2,3		LC
**Strigidae**			
*Athenecunicularia*	2,3	Pr	LC
**Order Piciformes**			
**Picidae**			
*Colaptesauratus*	2,3	E	LC
*Dryobatesscalaris*	2		LC
*Melanerpesuropygialis*	1		LC
**Order Falconiformes**			
**Falconidae**			
*Caracaracheriway*	1		LC
*Falcomexicanus*	2,3	A	LC
*Falcosparverius*	2,3		LC
**Order Passeriformes**			
**Tyrannidae**			
*Contopussordidulus*	3		LC
*Myiarchuscinerascens*	1,2,3		LC
*Pyrocephalusrubinus*	2,3		LC
*Tyrannusverticalis*	2		LC
*Sayornisnigricans*	2,3		LC
*Sayornissaya*	2,3		LC
**Laniidae**			
*Laniusludovicianus*	1,2,3		NT
**Corvidae**			
*Corvusbrachyrhynchos*	3		LC
*Corvuscorax*	2,3		LC
**Alaudidae**			
*Eremophilaalpestris*	3		LC
**Hirundinidae**			
*Stelgidopteryxserripennis*	2,3		LC
*Hirundorustica*	3		LC
*Petrochelidonpyrrhonota*	2,3		LC
*Tachycinetabicolor*	2,3		LC
**Remizidae**			
*Auriparusflaviceps*	1,2,3		LC
**Troglodytidae**			
*Campylorhynchusbrunneicapillus*	1,2,3		LC
*Cistothoruspalustris*	2,3		LC
*Salpinctesobsoletus*	1,2,3		LC
*Thryomanesbewickii*	2,3		LC
*Troglodytesaedon*	3		LC
**Polioptilidae**			
*Polioptilacaerulea*	2,3		LC
*Polioptilamelanura*	1,2,3		LC
**Regulidae**			
*Reguluscalendula*	2,3		LC
**Turdidae**			
*Catharusguttatus*	2		LC
*Catharusustulatus*	3		LC
**Mimidae**			
*Mimuspolyglottos*	1,2,3		LC
*Toxostomacrissale*	1		LC
**Sturnidae**			
Sturnusvulgaris*	2,3		LC
**Ptiliogonatidae**			
*Phainopeplanitens*	1,2,3		LC
**Passeridae**			
Passerdomesticus*	2		LC
**Motacillidae**			
*Anthusrubescens*	2		LC
**Fringillidae**			
*Haemorhousmexicanus*	1,2,3		LC
*Spinuspsaltria*	2,3		LC
**Passerellidae**			
*Amphispizabilineata*	1,2,3		LC
*Artemisiospizabelli*	3		LC
*Chondestesgrammacus*	2,3		LC
*Juncohyemalis*	1		LC
*Melospizalincolnii*	3		LC
*Melospizamelodia*	2,3		LC
*Melozoneaberti*	1,2,3		LC
*Pooecetesgramineus*	2		LC
*Spizellapasserina*	1,3		LC
*Spizellabreweri*	1,2,3		LC
*Zonotrichialeucophrys*	2,3		LC
**Icteridae**			
*Agelaiusphoeniceus*	1,2,3		LC
*Icterusbullockii*	3		LC
*Molothrusater*	1,2,3		LC
*Quiscalusmexicanus*	2,3		LC
*Sturnellaneglecta*	2,3		LC
*Xanthocephalusxanthocephalus*	3		LC
**Parulidae**			
*Geothlypistrichas*	2,3		LC
*Leiothlypiscelata*	2		LC
*Leiothlypisruficapilla*	3		LC
*Setophagacoronata*	1,2,3		LC
**Cardinalidae**			
*Passerinacaerulea*	3		LC
**Class Mammalia**			
**Order Didelphimorphia**			
**Didelphidae**			
*Didelphisvirginiana*	3		LC
**Order Lagomorpha**			
**Leporidae**			
*Sylvilagusaudubonii*	3		LC
*Lepuscalifornicus*	2,3		LC
**Order Rodentia**			
**Cricetidae**			
*Neotomalepida*	2,3		LC
*Peromyscuseremicus*	1,2		LC
*Peromyscusmaniculatus*	2,3		LC
*Peromyscuscrinitus*	1,2,3		LC
**Geomyidae**			
*Thomomysbottae*	3		LC
**Sciuridae**			
*Ammospermophilusleucurus*	1,3		LC
*Xerospermophilustereticaudus*	3		LC
**Heteromyidae**			
*Dipodomysdeserti*	1		LC
*Dipodomysmerriami*	1,2,3		LC
*Chaetodipusbaileyi*	1		LC
*Chaetodipusformosus*	2,3		LC
*Chaetodipuspenicillatus*	1,3		LC
*Chaetodipusspinatus*	1,2,3		LC
*Perognathuslongimembris*	3		LC
*Ondatrazibethicus*	3	A	LC
**Order Carnivora**			
**Mephitidae**			
*Mephitismephitis*	2		LC
**Canidae**			
*Canislatrans*	2,3		LC
*Urocyoncinereoargenteus*	3		LC
*Vulpesmacrotis*	3	A	LC
**Procyonidae**			
*Procyonlotor*	3		LC
**Felidae**			
*Lynxrufus*	3		LC
**Order Artiodactyla**			
**Bovidae**			
*Oviscanadensis*	1,2,3	Pr	LC
**Cervidae**			
*Odocoileushemionus*	2,3		LC
**Order Chiroptera**			
**Molossidae**			
*Tadaridabrasiliensis*	1,3		LC
*Eumopsperotis*	1,3		LC
**Vespertilionidae**			
*Eptesicusfuscus*	1		LC
*Macrotuscalifornicus*	1		LC
*Myotiscalifornicus*	3		LC
*Myotisvivesi*	1	P	VU
*Myotisyumanensis*	3		LC
*Parastrellushesperus*	1,3	A	LC
**Class Reptilia**			
**Order Squamata**			
**Suborder Lacertilia**			
**Phrynosomatidae**			
*Callisaurusdraconoides*	1,2,3		LC
*Crotaphytusgrismeri*	1,3		LC
*Dipsosaurusdorsalis*	3		LC
*Phrynosomaplatyrhinos*	3		LC
*Sauromalusater*	3	Pr	LC
*Sceloporusmagister*	1,3		LC
*Urosaurusgraciosus*	3		LC
*Urosaurusornatus*	3		LC
*Utastansburiana*	1,2,3	A	LC
**Teiidae**			
*Aspidoscelistigris*	1,2,3		LC
**Suborder Serpentes**			
**Colubridae**			
*Masticophisflagellum*	1	A	LC
**Natricidae**			
*Thamnophismarcianus*	1	A	LC
**Viperidae**			
*Crotaluscerastes*	1,2	Pr	LC
*Crotalusmitchellii*	1	Pr	LC

In general, data sources contributed with multiple overlapping species (i.e. same species reported in two or more data sources), but each source also contributed with unique species (Table 1). Of the 102 species of birds, we recorded 79 during fieldwork and 20 of them were observed exclusively with this method; similarly, we obtained records of 23 bird species from GBIF and five were not recorded in any other data source; and citizen science platforms contained records for 76 species, 19 of which were only reported there. In the case of mammals, we recorded 27 species during fieldwork, with 12 of them observed exclusively with field efforts; similarly, GBIF and iNaturalist contained records for 15 and 12 species respectively, five and one of which were only recorded respectively in each platform. For reptiles, five of the 10 species that we identified during fieldwork were not reported in other data source; however, three out of the nine species obtained from GBIF were not reported in any other data source. The four reptile species reported in citizen science platforms from Cucapá were either observed in the field or reported in GBIF.

In terms of number of records and locations, birds are the best represented group in the study area, followed by mammals, while reptiles are the group with the fewest records. The records are located mainly in the south, southeast, and central-west parts of the study area, covering the entire elevation range of Cucapá, while wide high areas with rugged terrain in the western portion, the northern area, and some low and flat areas to the east lack records (Fig. 1).

According to SEMARNAT (2010), 21 of the vertebrate species reported here are included in some risk of extinction category. In the case of birds, two species are Threatened, seven are Subject to Special Protection, and one is Probably Extinct in the Wild. For mammals, two species are Threatened, one is Subject to Special Protection, and one is Endangered. In the case of reptile species, four are Threatened and three are Subject to Special Protection. According to the IUCN Red List, only one species of mammal is Vulnerable (Table 1).

### ﻿Comparison of species richness and composition with natural protected areas

Bird species richness in Cucapá (102 spp.) is higher than richness in community NPA Rancho Rodeo del Rey (90 spp.), but lower than in the rest of the NPAs. Mammal species richness in Cucapá (34 spp.) is higher than in HDRC (30 spp.) and SHRRC (24 spp.), but lower than in the rest of the NPAs. For reptiles, species richness in Cucapá (14 spp.) was lower than for all NPAs (Table 2). The largest differences in bird species composition were between the community NPA Rodeo del Rey and RBAGC, Constitución de 1857, and Laguna Hanson (β_jac_ = 0.72 in all cases; Table 3A); for mammal and reptile species, the largest differences were between Laguna Hanson and HDRC (β_jac_ = 0.81 and 0.89 respectively; Table 3B, C). The lowest total difference in species composition of birds, mammals and reptiles were between Laguna Hanson and Constitución de 1857 NPA (β_jac_ = 0.08, 0.42 and 0.34, respectively; Table 3A–C).

**Table 2.** Number of orders, families, genera, and species of birds, mammals and reptiles recorded in Sierra Cucapá and six NPAs of northern Baja California: Parque Nacional Constitución de 1857, Reserva de la Biosfera del Alto Golfo de California (RBAGC), Humedales Remanentes del Río Colorado (HDRC), Sistema de Humedales Remanentes del Río Colorado (SHRRC), Laguna Hanson, Rodeo del Rey (community).

NPA/region	Taxonomic group	Orders	Families	Genera	Species
**Sierra Cucapá**	Birds	15	38	83	102
	Mammals	6	14	26	34
	Reptiles	1	5	12	14
**Constitución 1857**	Birds	18	45	116	174
	Mammals	8	19	41	63
	Reptiles	1	15	30	48
**RBAGC**	Birds	22	58	174	320
	Mammals	8	21	42	58
	Reptiles	2	13	32	46
**HDRC**	Birds	20	50	139	217
	Mammals	7	13	26	30
	Reptiles	2	10	17	21
**SHRRC**	Birds	20	49	145	241
	Mammals	6	10	18	24
	Reptiles	1	12	25	28
**Laguna Hanson**	Birds	18	44	110	165
	Mammals	7	15	26	39
	Reptiles	1	12	20	31
**Rodeo del Rey (C)**	Birds	15	36	68	90

**Figure 2.**
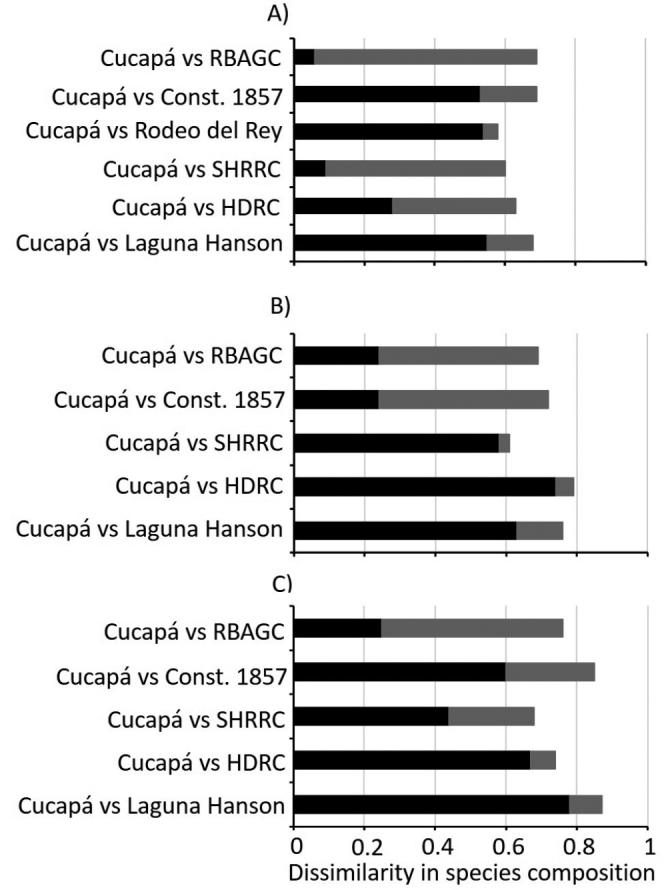
Differences in species composition between Cucapá and the NPAs**A** birds **B** mammals **C** reptiles. Total difference in species composition (β_jac_; complete bar), proportion of the difference due to species turnover (β_jtu_; black portion of bar) and proportion of the difference due to nestedness (β_jne_; gray portion of bar).

Total differences in species composition (β_jac_) between Cucapá and the NPAs ranged between 0.58–0.69 for birds (Table 3A; Fig. 2A), 0.61–0.79 for mammals (Table 3B; Fig. 2B), and 0.69–0.87 for reptiles (Table 3C; Fig. 2C). The differences in species composition between Cucapá and the NPAs were mainly explained by turnover of species in the comparisons with Laguna Hanson, Rodeo del Rey and Constitución 1857, in the case of birds (β_jtu_ = 0.55, 0.54, 0.53, respectively; Table 3A; Fig. 2A), for mammals in the comparisons with Laguna Hanson, HDRC and SHRRC (β_jtu_ = 0.62, 0.74, 0.58, respectively; Table 3B; Fig. 2B), and for reptiles in the comparisons with Laguna Hanson, HDRC, SHRRC, and Constitución 1857 (β_jtu_ = 0.78, 0.67, 0.44 and 0.60, respectively; Table 3C; Fig. 2C). In contrast, the differences in composition were mainly explained by nestedness in the comparisons between Cucapá and HDRC, SHRRC, and RBAGC for birds (β_jne_ = 0.35, 0.51, 0.63, respectively; Table 3A; Fig. 2A), for mammals in the comparisons between Cucapá with Constitución 1857 and RBAGC (β_jne_ = 0.48, 0.45 respectively; Table 3B; Fig. 2B) and for reptiles only in the comparison between Cucapá and RBAGC (β_jne_ = 0.51; Table 3C; Fig. 2C).

**Table 3.** Differences in species composition between pairs of sites. A birds B mammals C reptiles. The values outside the parentheses correspond to the total difference in species composition (β_jac_), the first value inside the parentheses indicates the difference due to species turnover (β_jtu_) and the second value indicates the proportion due to nestedness (β_jne_).

	Sierra Cucapá	Laguna Hanson	HDRC	SHRRC	Const. 1857	RBAGC
**A)**						
**Laguna Hanson**	0.68 (0.55+0.13)					
**HDRC**	0.63 (0.28+0.35)	0.62 (0.53+0.09)				
**SHRRC**	0.60 (0.09+0.51)	0.59 (0.44+0.15)	0.31 (0.25+0.06)			
**Const. 1857**	0.69 (0.53+0.16)	0.08 (0.02+0.06)	0.61 (0.54+0.07)	0.56 (0.44+0.13)		
**RBAGC**	0.69 (0.06+0.63)	0.61 (0.29+0.32)	0.33 (0.02+0.31)	0.33(0.12+0.21)	0.60 (0.31+0.29)	
**Rodeo del Rey**	0.58 (0.54+0.04)	0.72 (0.55+0.17)	0.58 (0.02+0.56)	0.64 (0.08+0.56)	0.72 (0.53+0.19)	0.72 (0.02+0.70)
**B)**						
**Laguna Hanson**	0.75 (0.62+0.13)					
**HDRC**	0.79 (0.74+0.05)	0.81 (0.78+0.03)				
**SHRRC**	0.61 (0.58+0.03)	0.79 (0.70+0.09)	0.65 (0.59+0.06)			
**Const. 1857**	0.72 (0.24+0.48)	0.42 (0.05+0.37)	0.76 (0.57+0.19)	0.72 (0.34+0.38)		
**RBAGC**	0.69 (0.24+0.45)	0.78 (0.70+0.08)	0.54 (0.12+0.42)	0.64 (0.15+0.49)	0.59 (0.56+0.03)	
**C)**						
**Laguna Hanson**	0.87 (0.78+0.09)					
**HDRC**	0.74 (0.67+0.07)	0.89 (0.86+0.03)				
**SHRRC**	0.69 (0.44+0.24)	0.77 (0.75+0.02)	0.63 (0.52+0.11)			
**Const. 1857**	0.85 (0.60+0.25)	0.34 (0+0.34)	0.84 (0.71+0.13)	0.63 (0.44+0.19)		
**RBAGC**	0.76 (0.25+0.51)	0.83 (0.78+0.05)	0.64 (0.18+0.46)	0.62 (0.40+0.22)	0.70 (0.69+0.01)	

## ﻿Discussion

To our knowledge, this is the first study that integrates information on the diversity of terrestrial vertebrates that inhabit Cucapá and analyzes its conservation value. Our results indicate that Cucapá is home to a considerable number of bird, mammal, and reptile species. The number of species recorded in Cucapá represent 22% of the 473 bird species (Erickson et al. 2013), 48.5% of the 70 species of mammals (Guevara-Carrizales et al. 2016), and 14% of the 99 reptile species (Hollingsworth et al. 2015) reported from Baja California state. These percentages take on a greater importance considering that Cucapá occupies only 0.5% of the state.

The geographic distribution of bird, mammal, and reptile records available through GBIF, iNaturalist, and eBird indicates that large areas of Cucapá remain unexplored. Furthermore, some nearby NPAs (e.g. Parque Nacional Constitución de 1857 and Laguna Hanson) hold greater richness in considerably smaller areas. Thus, we consider that species richness of terrestrial vertebrates in Cucapá might actually be higher than what we report here, especially considering that at least 41, five, and 14 additional species of birds, mammals, and reptiles respectively have been recorded in nearby locations (Hathaway 2000; Guevara-Carrizales et al. 2013) and could inhabit Cucapá. Further efforts in unexplored areas of Cucapá are needed to complement the biological inventory of terrestrial vertebrates presented here. Population-level studies in the region, especially of threatened and endangered species, would be desirable to deepen our understanding of the conservation value of this region. Considering that the lowest elevation of Cucapá is essentially at sea level, future studies could consider climate change predictions in order to evaluate the persistence of species under future scenarios (e.g. with reptiles, see Lara-Reséndiz et al. 2020; Pérez-Delgadillo et al. 2021).

The inclusion of records from three different data sources provided complementary contributions to the inventory of Cucapá. The high percentage of bird, mammal, and reptile species recorded during fieldwork (78%, 79%, and 71%, respectively) and the percentage of species recorded exclusively by this method (20% for birds, 35% for mammals, and 35.7% for reptiles) indicate the importance of field sampling efforts to the species inventory for this relatively unexplored area. It also suggests the need for further fieldwork in areas that still lack information, but seasonal variations in species composition and overall activity should be considered. In the case of birds, for instance, seasonal changes are particularly marked due to migratory processes (Danemann et al. 2002). The contribution of records from citizen science platforms was notable for birds, with 76% of all species that we recorded being reported there, and 18% of species recorded exclusively with this data source. This demonstrates the utility of such platforms for studies that integrate knowledge of species diversity and that evaluate the conservation value of areas that are poorly explored and of which little is known of their flora and fauna (Schmiedel et al. 2016). The effectiveness and precision of birdwatchers, for example on the eBird platform, have been linked to the experience and high level of interest in the group, since the identification skills of the participants have been inferred to be as good as those of ornithologists with scientific training (Lukyanenko et al. 2016).

The number of species listed in the NOM-059 (Table 1) highlights the value of Cucapá as an important area in the region for the conservation of terrestrial vertebrates. In the case of birds, it is particularly important for the presence of raptor species threatened with extinction (genera *Accipiter*, *Buteo*, *Parabuteo*, and *Athene*), and of species considered threatened throughout North America such as *Athenecunicularia* and *Laniusludovicianus* (CCA 2000; SEMARNAT 2010), since several of these migrant species require natural areas along their migration route. For mammals, it is worth highlighting the presence in Cucapá of threatened species that need large areas of land due to their environmental requirements, such as *Oviscanadensis* (SEMARNAT 2010), which is considered a flagship species in the arid-mountainous ecosystems of Mexico (SPABC 2012) and whose Peninsular subspecies (*Oviscanadensisnelsoni*) is considered Endangered in the USA according to the U.S. Fish and Wildlife Service (https://ecos.fws.gov/ecp/species/4970). Cucapá is thus important for the conservation of this species in situ because of the extension of habitat it conserves and because it connects parts of the Sistema de Humedales Remanentes del Río Colorado located to the west and east of Cucapá (Fig. 1). In the case of reptiles, besides the presence of four Threatened and three Subject to Special Protection species, there is one particular species not listed neither in the IUCN or NOM-059 which we consider makes Cucapá a priority area for conservation. This is *Crotaphytusgrismeri*, a lizard endemic to Cucapá and Sierra El Mayor (McGuire 1994), which is another unprotected mountainous area south of Cucapá. Therefore, *C.grismeri* is not included in any NPA (Ramírez-Acosta et al. 2012). This illustrates that there is no need to wait until a species with particular ecological requirements is considered to be under a risk category to start protecting it.

The diversity of vertebrates and the number of species under risk categories reported in this study suggest that Cucapá represents an important conservation region and an opportunity for biological conservation in the northern Peninsula. Currently, Cucapá is not under any legal protection scheme and is therefore not within the NPAs system, not even within the priority terrestrial regions of the state of Baja California (Arriaga et al. 2000a, 2000b) or any other initiative that would provide for its protection in the future. Both in the interior and in the surroundings of the Sierra, various anthropogenic activities threaten its biodiversity (e.g. mining, agriculture, off-road racing, and illegal looting of species for sale; Mellink 1995). Additionally, the recently approved Proyecto Integral EcoZoneMX will allow activities that include mining, commercial construction, and a photovoltaic farm in an area of 14,782 ha (VRM 2015) that overlaps with more than 40% of Cucapá, which would have considerable negative effects on its biodiversity.

### ﻿Comparison of species richness and composition with NPAs

For the three groups of vertebrates evaluated here, species richness in Cucapá is not higher than in the nearby NPAs with biological information (Table 2). However, we found that the difference in species composition between Cucapá and the NPAs is greater even than the differences between pairs of NPAs and, for each group, we also found a high species turnover between Cucapá and three or more NPAs (Table 3). This indicates that Cucapá offers a complementary conservation value to the NPAs system in the northern Peninsula. Thanks to its location, Cucapá can forms a biological corridor in the northern Peninsula in conjunction with the closest NPAs in the region (RBAGC, HDRC, and SHRRC), and with other unprotected natural areas such as Sierra El Mayor. If Cucapá were protected, this corridor would favor 1) the conservation of resident species populations (particularly those with small distribution ranges or that are endemic to this region, e.g. *Crotaphytusgrismeri*), and 2) the transit of migrants (e.g. birds and certain species of bats), and of mammalian species with wide distribution ranges (e.g. bighorn sheep and mule deer).

Several studies have evaluated the role that the NPAs system in the northern Peninsula has for the conservation of biodiversity in the state of Baja California (Riemann and Ezcurra 2005; Lovich et al. 2009; Ramírez-Acosta et al. 2012) and north of it (White et al. 2006; Barrows et al. 2013). These studies, however, did not include, or included tangentially, Ramsar sites, private or community NPAs, or natural areas that are not formally protected, such as Cucapá. This is possibly due to a lack of information about the biological diversity that inhabits these spaces. For example, for the private NPA Rancho Rodeo del Rey and the community NPA El Doctor, we did not find species records for any of the vertebrate groups evaluated here (Table 2). Therefore, we recommend further studies to collect biological information for these sites. Considering that this region is subject to multiple threats as habitat modification, pollution, introduction of exotic species, and other processes that are affecting ecosystems (Valdés-Casillas et al. 1998; Quintero-Núñez and Moncada-Aguilar 2008; Rosete-Vergés et al. 2008; Andreu-Soler et al. 2014), we consider crucial that all NPAs are included in future evaluations of the conservation potential in the northern Peninsula, regardless of their type of governance. Other studies have shown that non-government NPAs can have considerable complementarity with government NPAs, and that they contribute to secure the protection of a higher proportion of biodiversity (Ochoa-Ochoa et al. 2009; Muench and Martínez-Ramos 2016; García-Bañuelos et al. 2020).

The current situation of Cucapá should draw attention of the urgent need to implement strategies for the conservation of its biodiversity. Among the activities that have been effective at slowing the deterioration of other unprotected areas with high biological diversity are the establishment of NPAs under non-government administration. Some examples include the Wildlife Management and Sustainable Use Units (UMA from its name in Spanish; Gallina-Tessaro et al. 2009; Pozo-Montuy et al. 2017), private NPAs (Ortiz-Lozada et al. 2017; Villegas-Patraca et al. 2020), and community NPAs (Urquiza-Haas et al. 2011). It would be desirable to promote these alternatives for Cucapá.

## ﻿Conclusions

We found that Cucapá harbors high bird, mammal, and reptile species richness. Considering the number and size of unexplored areas, as well as the cryptic behavior and generally low detection probabilities of some species or groups (e.g. reptiles), species richness could be even higher than what we report here. The Cucapá Sierra has a particular species composition of the three groups of vertebrates, with high species turnover with three or more NPAs, and it harbors several species in risk extinction categories as well as endemic species. Taken together, these characteristics indicate that Cucapá has a complementary conservation value to the NPAs system of a region that has been severely transformed by various human activities. The lack of records in certain areas indicates the need to carry out further fieldwork to complement the species inventory reported here. In terms of conservation, we recommend the urgent establishment of strategies for the protection of Cucapá and its biodiversity.
